# Micronutrient deficiency and supplements in schoolchildren and teenagers

**DOI:** 10.1097/MCO.0000000000001027

**Published:** 2024-03-08

**Authors:** Mette M. Berger, Alan Shenkin

**Affiliations:** a Faculty of Biology & Medicine, Lausanne University, Lausanne, Switzerland; b Institute of Aging and Chronic Disease, University of Liverpool, Liverpool, UK

**Keywords:** adolescent, anaemia, attention-deficit / hyperactivity disorder, iron, nutrition, podiatry, vitamin D

## Abstract

**Purpose of review:**

The essential micronutrients are corner stones in the functional and physical development. Early deficiency has life-long consequences. While awareness about iron deficiency is relatively high, it remains lower for other micronutrients. This review aims at reporting on recent data and attracting attention to the high prevalence of micronutrient deficiencies in school-age and adolescent individuals.

**Recent findings:**

Iron deficiency anaemia remains highly prevalent worldwide and the most frequent deficiency but can be corrected with simple tools ranging from food fortification, nutritional intervention, and to supplements. The link between micronutrient (MN) deficiency and neurobehavioral disorders is increasingly established and is worrying even in Western countries. Paediatric individuals are prone to imbalanced diets and picky eating behaviour, and their diets may then become incomplete: the highest risk for deficiency is observed for iron, zinc and vitamin D.

**Summary:**

There is not much new information, but rather confirmation of the importance of health policies. Well conducted randomized controlled trials confirm that deficiencies can be corrected efficiently including with food fortification, and result in clinical benefits. Individual complementation should be considered in children and adolescents with proven deficiency.

## INTRODUCTION

The daily requirements for micronutrients in the paediatric population have been well described in international recommendations. In the USA, the Dietary Reference Intakes (DRI) for the paediatric population were published in 2011 a revision of, including the tolerable upper intake level values [1], while in the European Food Safety Authority (EFSA) actualized the values in 2017 [2]. Covering the DRI with oral feeding is paradoxically difficult in numerous children and adolescents compared to those depending on parenteral nutrition: indeed many of them do not consume ‘adequate’, balanced food, that is do not cover their DRI for multiple different reasons, including the high proportion of children with picky eating behaviour, largely a phenomenon of industrialized countries that stresses parents, but also poor diets in adolescents [3]. 

**Box 1:**
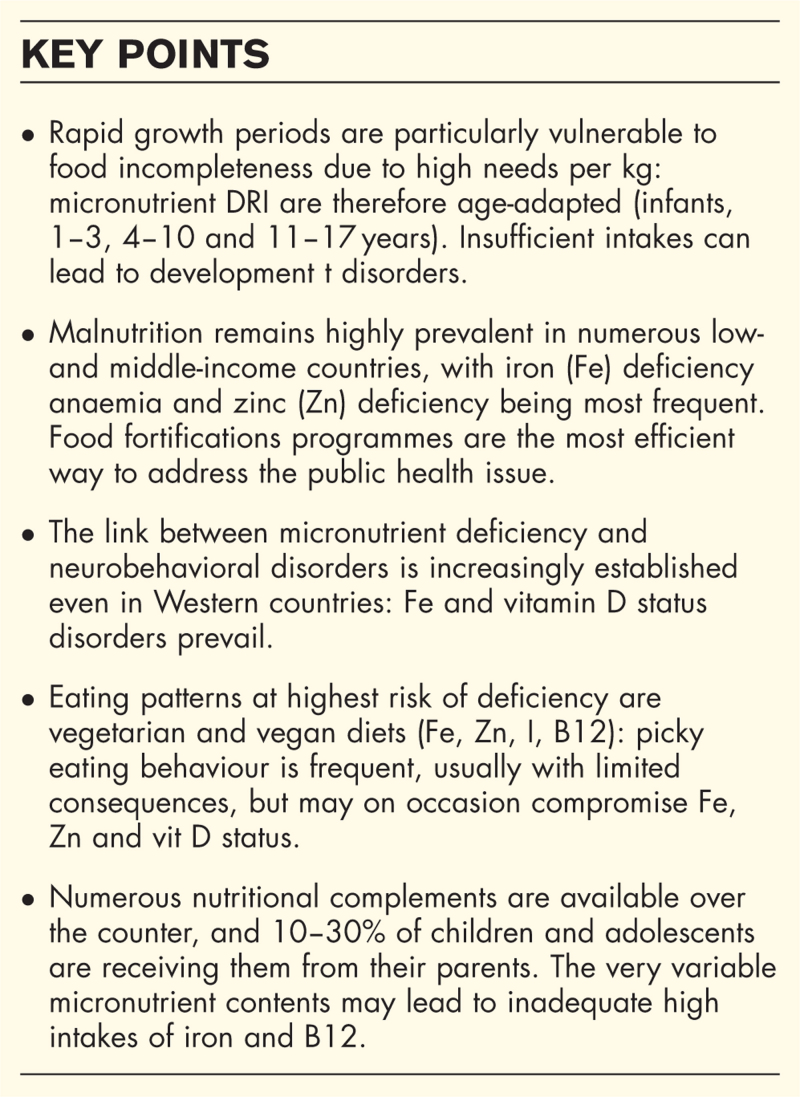
no caption available

Nevertheless, there are situations in which complementation or supplementation might be considered, to either complete a micronutrients poor diet, or treat a deficiency. In his editorial, Conrad Cole noted that micronutrients malnutrition can be referred to as a ‘hidden hunger’ [7], that may occur in the presence of an otherwise nutritionally or energy appropriate diet. It can be caused by eating food that is cheap and filling, but deficient in essential micronutrients, which is more likely in periods of economic depression [7]. A large German cohort study showed that most children were covering their needs, with iodine, iron and vitamin D being frequently at the lower limit [8]. Which children should benefit from a more advanced diagnosis of their micronutrient status, and eventually receive supplements?

For paediatric patients depending on parenteral nutrition, nutritional guidelines provide exact recommendations [9], which have recently been re-updated [10]. As in adults on medical nutrition therapy, monitoring blood levels is recommended as underlying conditions may modify the needs [10], while the one-size-fits-all complement products may not cover specific needs [11].

Micronutrients deficiency generally is associated with global insufficient feeding, i.e. malnutrition, but with regional specificities [4]. Studies conducted under the authority of World Health Organization [5] and UNICEF [6] have shown the efficacy of food fortification programs. UNICEF has listed the critical micronutrients associated with public health problems, that is, those for which deficiency hinders development and function, including vitamin A (blindness), iron (anaemia), iodine (goitre and brain development) and folic acid (in adolescent girls) [12]. But other micronutrients are highly susceptible to deficiency with deleterious health consequences, such as zinc (immunity), vitamin D (bone health, rickets) and vitamin C (scurvy). Table 1 provides a summary of functions and clinical symptoms with their DRI by age category of the above MNs. In any country characterized by endemic micronutrient deficiencies, this should be specifically addressed with dedicated food fortification policies, as no individual micronutrient administration would be able to achieve equivalent results.

**Table 1:** Functions, clinical symptoms in children and DRI for the most frequently deficient micronutrients [1,11]

Micronutrient	Principal functions	Clinical symptoms of deficiency	Age (years)	DRI
Iodine (μg/day)	Constituent of thyroid hormones and a regulator of thyroid gland function	Goitre, hypothyroidism, cretinism	4–8 9–13 M+F 14–18 M+F	65 73 95
Iron (mg/day)^a^	functional component of heme, oxygen binding and transport (haemoglobin, myoglobin), oxygen metabolism (catalases, peroxidases), cellular respiration and electron transport (cytochromes). DNA synthesis, gene regulation, drug metabolism and steroid synthesis	Anaemia, impaired physical and cognitive function, immunity alteration	4–8 9–13 M+F 14–18 M 14–18 F	10 8 11 15
Selenium (μg/day)	component of at least 25 selenoproteins: biochemical functions include antioxidant and redox activity, control of thyroid hormone metabolism	Pale nails, Muscle weakness, Fatigue, Hair loss, Weakened immune system, Kashin Beck osteochondropathy, Keshan cardiomyopathy, depressed immunity	4–8 9–13 M+F 14–18 M+F	23 35 45
Zinc (mg/day)	The numerous zinc metalloenzymes play essential roles in virtually all metabolic pathways. Zinc has structural, catalytic and regulatory functions.	growth retardation, delayed sexual development and bone maturation, alopecia, skin rash of face, groins, hands and feet impaired wound healing and immune function, diarrhoea and blunting of taste and smell	4–8 9–13 M+F 14–18 M 14–18 F	4,0 7,0 8,8 7,3
Vitamin A (μg/day) (retinol equivalent)	Prohormone with active metabolites. ligands for nuclear receptors (RAR, RXR, PPARs), which activate gene expression in more than 500 target genes, important role in the immune system	Vision loss or blindness, Dry, itchy, scaly skin. Delayed growth and development in children, Respiratory tract infections	4–8 9–13 M 9–13 F 14–18 M 14–18 F	275 445 420 630 485
B9 folic acid μg/day (dietary folate equivalents)	Folate, as tetrahydrofolate, is required for the body to make DNA and RNA and metabolise amino acids. One-carbon metabolism: in cytoplasm, is required for the synthesis of purines and thymidylate, and remethylation of homocysteine to methionine; in mitochondria is required for the synthesis of formylated methionyl-tRNA	Most symptoms of folate deficiency overlap with cobalamin deficiency, i.e. megaloblastic anaemia and pancytopenia, glossitis, angular stomatitis, oral ulcers, neuropsychiatric manifestations, including depression, irritability, insomnia, cognitive impairment, psychosis, anorexia and fatigue	4–8 9–13 M+F 14–18 M+F	160 250 330
B12 Cobalamin (μg/day)	Cofactor for two enzymes: methionine synthase in methyl transfer from methyl tetrahydrofolate to form methionine from homocysteine; and methyl malonyl-CoA mutase in synthesis of the citric acid cycle intermediate succinyl CoA. Essential for mitochondrial metabolism, immune response, preservation of DNA integrity and neuronal myelin sheath, and synthesis of neurotransmitters	Anaemia, vomiting, lethargy, failure to thrive, hypotonia, headaches, indigestion loss of appetite, exhaustion, diarrhoea a sore tongue, mouth ulcers cognitive changes, muscle weakness	4–8 9–13 M+F 14–18 M+F	1,0 1,5 2,0
Vitamin C (mg/day)	numerous functions based on electron donation, potent water-soluble antioxidant, cofactor/co-substrate for the biosynthesis of neurotransmitters (noradrenaline, serotonin), cortisol, peptide hormones (vasopressin), and collagen	Scurvy, gingival haemorrhage, gingivitis, petechiae, rash, internal bleeding, impaired wound healing, impaired growth	4–8 9–13 M+F 14–18 M 14–18 F	22 39 63 56
Vitamin D^b^ (μg/day) cholecalciferol	steroid hormone precursor involved in the regulation of numerous genes, receptor is expressed in many tissues, including muscle (skeletal and cardiac), bone, immune system, skin and endocrine organs	Rickets, altered immune system, muscle, heart, and nervous system/brain	4–8 9–13 M+F 14–18 M+F	5 5 5

a For iron, the doses are RDA: as only a fraction of dietary iron is absorbed, the dietary requirement is considerably higher than the net absorbed iron requirement.

b For cholecalciferol the doses are RDA (not DRI) (conversion: 1 μg cholecalciferol = 40 IU).

The aim of this review is to attract attention to the frequency of insufficient or deficient micronutrient status in the paediatric population, focusing on school-age children and adolescents since the consequences of micronutrient deficiencies might be more readily observable in this age group.

## MATERIALS AND METHODS

The present review focuses on studies investigating school-age children and adolescents up to 20 years, excluding infants and toddlers. Search words were ‘micronutrient supplement’ (vitamin or trace element), ‘Micronutrient deficiency’, ‘Malnutrition’, ‘Clinical trials’ AND ‘Schoolchild or adolescent’ published during the last 4 years with special attention to the last two years.

## MICRONUTRIENT DEFICIENCIES

Deficiency is defined as an intake below the standard recommendation and either the presence of clinical signs or symptoms or blood/plasma concentrations below reference range together with metabolic effects of inadequacy [11]. Due to growth, children have a higher nutrient requirement per kilogram of body weight than adults, and insufficient intakes may lead to disorders. The proportion of children and adolescents with poor diets is high worldwide, and many young children are not meeting RDI for several micronutrients [13]. Deficiencies, whether diseases specific or due to global malnutrition, will have negative short-term and long-term effects, including physical, developmental and cognitive impairment, increased susceptibility to infections, higher morbidity and mortality, and decreased productivity later in life [14]. Typically, zinc deficiency during childhood was shown by Prasad in the 60 s to cause growth retardation [15]. In the 90 s, zinc supplements combined with/without other MNs (mainly iron) were recognized to be beneficial in the presence of a diet low in animal products and based on high-phytate cereals and legumes (especially during the period of complementary feeding), together with severe growth stunting, low plasma zinc concentrations and episodes of persistent diarrhoea [15].

The early correction of deficiency may be of major significance, as deficiency can impact cognitive function in later life, as shown by a follow-up study from the Santiago area (Chile): 1018 adolescents underwent psychological evaluation, and individuals who were enrolled were ‘normal’ at birth but had iron deficiency diagnosed between 12 and 18 months of age [16]: participants who had received iron supplementation in infancy had lower parent-reported conduct disorder symptoms than those who did not. The threshold was ‘deficiency’ with or without anaemia [16]. Infancy iron deficiency seems associated with social problems in adolescence: the prevention opportunity looks of utmost human and social importance.

Vitamin C deficiency can result in scurvy, which is generally considered rare in high-income countries, but it has increasingly been reported [17^▪^]: a high level of suspicion should probably be maintained in children with musculoskeletal complaints.

The risk of focusing on single micronutrients rather than on the bundle of essential micronutrients incurs the risk of overlooking other micronutrient inadequacies. Nutrition-focused strategies should therefore have a major place in handling these widespread problems [18^▪^].

The above mentioned picky-eating behaviour is present in countries with or without food-insecurity. This usually resolves without affecting the child's development, but there is a subgroup of children for whom this is not the case [3]. There is a consensus for a risk of insufficient iron and zinc intake [3,19^▪▪^]. A Taiwanese study including 203 children detected picky eating in 67.5% of children in whom a high prevalence of low zinc levels was associated with low development and poor physical activity [19^▪▪^]: while not adjusted for confounders, this study should be considered a warning sign. This category of children merits close monitoring, and eventually more advanced diagnosis of status if deviations from the standard percentiles is detected.

## RECENT INTERVENTION STUDIES

Several studies have tested micronutrient complements and supplements in conditions associated with micronutrient deficiency such as malnutrition, anaemia, psychological disorders and other diseases.

### Malnutrition

Malnutrition remains widespread and includes micronutrient deficiencies. Poor diets are commonly present in adolescents. This pivotal period of life, which is frequently associated with some major changes in eating habits, makes adolescents a vulnerable group to commercial exploitation and other unhealthy influences, with lifelong and intergenerational consequences [18^▪^]: it may result in both underweight and overweight development [3].

Multiple micronutrient deficiencies exist in schoolchildren in countries like India. The gap between nutrient intake and requirements may be bridged by fortification, enabling fighting deficiencies as shown by Kuriyan *et al.*[20] who used a multi-micronutrient fortified milk beverage 6 days/week for 5 months. The prevalence of iron deficiency and vitamin B12 deficiency decreased significantly with no major effect on cognitive or physical performance [20]. The global malnutrition issue should ideally be corrected first.

A high energy intake RCT tested a daily lipid-based 500 kcal supplement in 34 malnourished children aged 7.1 ± 1.5 years and conducted over 4 weeks [21]: anthropometric variables, haemoglobin (Hb) and iron levels (*P* < 0.001) improved.

Endemic selenium deficiency affects some specific geographic areas distributed in different countries over the globe. In an area with a high prevalence of Kashin-Beck disease, a degenerative cartilage disorder [22^▪^], a Chinese study including 240 children showed that the dietary Se intake was 40.0% in the endemic area. The study also showed that low diversity of food was contributive, opening the type of intervention to more than only selenium fortification.

Several case reports (not referenced herein) show that imbalanced diet resulting from exclusive consumption by children and adolescents of junk food and snacks can result in severe selenium, vitamin C and vitamin A deficiencies, and may even result in dramatic clinical pictures, such as acute blindness.

Lacto-ovo-vegetarian diets are generally well balanced if they include sufficient amounts of dairy products [23^▪^]. But this is not the case with vegetarian, and especially strict vegan diets: iron, zinc, iodine, and vitamins B12 and D intakes are usually below DRI, as are the intakes of omega-3 PUFA. The risk of iodine deficiency is particularly high in vegetarian/strict vegan children as shown by a recent Czech study that included an investigation of thyroid function [24^▪^]. The same group studied vitamin B12 status; vegetarian/strict vegan children and observed a high prevalence of over-supplementation with high blood levels [25]: the impact of persistent B12 hypervitaminosis deserves future studies. Some national guidelines already exist for this category of children [26], and will certainly be completed in the future. Performing appropriate blood tests enables diagnosis of deficiency (or excess) [26], and prescription of adapted food complements during rapid growth periods limits the risk of deficiency [23^▪^].

### Anaemia – iron, B9, B12 deficiency

Anaemia adversely affects cognitive and motor development and causes fatigue and low productivity: it remains disproportionally prevalent in low and middle-income countries, mostly affecting women and children [27]. Iron deficiency and anaemia undermine childhood development, leading to poor cognitive and physical performance and increased susceptibility to disease. A study including data from 107 countries from 1995 to 2011, showed a modest improvement of Hb over time, reflecting intervention policies, with a prevalence of anaemia in children decreasing from 48 to 43%, still translating into 273 million anaemic children [27]. Similarly, among adolescents, anaemia is a leading cause of morbidity and mortality with long-term health and economic consequences [28^▪^]. A cross-sectional survey conducted among 2479 secondary school students in Zanzibar to determine the prevalence and factors associated with anaemia showed that 53.3% were anaemic according to WHO criteria: women had higher odds ratio (OR) of anaemia than men (OR = 1.47), as had stunted adolescents (OR = 1.38), and those without access to private toilets (OR = 1.68): these results confirm the importance of sanitation, hygiene and nutritional measures (iron-rich foods), that is a multifaceted intervention.

Hereafter, some recent RCTs have shown that the impact of iron deficiency can be reduced by different strategies. A community based RCT conducted in 226 Ethiopian girls tested a 3-month trial of weekly iron+folic acid supplements [29]. Initially, about 49% of adolescents had marginal iron stores, and significant improvement of serum folate, serum ferritin and Hb concentrations was observed compared to control. A RCT testing daily supplements of iron+folic acid with/without vitamin B_12_ for 90 days in 760 adolescent anaemic Indian girls showed correction of iron status and of vitamin B_12_ deficiency, the addition of B_12_ having no significant effect [30^▪^]. A RCT investigating a multi-micronutrient supplement containing 10 mg iron, 10 mg zinc and 400 μg vitamin A in 347 Vietnamese school children over 22 weeks [31^▪^] showed a significant increase of the mean corpuscular volume (MCV), serum ferritin, plasma zinc and plasma retinol. A RCT comparing parenteral (IM or IV) and oral B12 1000 ug supplements in 80 deficient anaemic children [32] showed that the parenteral route was more efficient in raising B12 blood levels (*P* = 0.016) and increasing Hb levels (*P* = 0.001) [32]. Of note, 55% were following a vegetarian diet and malnutrition was present in one-third of the children.

Enriching meals is effective. A small RCT including 22 preschool children (3 years old) testing a multi-micronutrient powder to enrich meals showed a significant reduction of anaemia and iron deficiency compared with placebo, improved language and inhibitory control and reduced developmental disparities [33^▪▪^]. A RCT conducted in 60 Egyptian kindergarten and primary school children tested the capacity of ready to eat fried liver meat balls (LMB) to fight anaemia and vitamin A deficiency and promote cognitive function in for 90 days: 43% of children were initially anaemic [34^▪▪^]. The LMB contributed to significant increases in the intakes of highly bioavailable Fe and vitamin A in the diets of all children, with an increase in height-for-age *Z* score and blood Hb. The standard scores of verbal and nonverbal cognitive function tests increased significantly (*P* < 0.05) with LMB. This study shows that simple effective and sustainable interventions can be developed.

Reducing iron deficiency has obvious beneficial effects on neurodevelopment, but iron overload may have adverse functional effects (intestinal and neurological impact) [35]. Respecting DRIs is particularly important in infants [35] as is prevention of intoxications that may occur through accidental intakes of candylike adult iron products [36].

### Vitamin D deficiency

As in adults, vitamin D deficiency is common in children. Effectiveness of complements/supplements in improving blood levels of vitamin D does not necessarily translate into improvement in function.

A secondary analysis of a RCT conducted in Mongolia in 8851 school-aged children (mean age 9.4 years) living in a setting where vitamin D deficiency is highly prevalent, testing weekly oral doses of 14 000 IU vitamin D3 for 3 years, showed that 95.5% were deficient at baseline. The intervention was effective in elevating 25(OH)D blood concentrations but did not influence growth, nor self-assessed pubertal development [37^▪^].

In tuberculosis (TB) in Indonesia teenagers, a RCT testing 1000 IU/day for 6 months in 80 Vitamin D deficient children with newly diagnosed TB showed a significantly (*P* < 0.001) faster resolution of fever, cough, improved malnutrition status and higher vitamin D level in the intervention group [38^▪^].

In US children with persistent asthma, a RCT including 192 children (mean age 9.8 years) showed no effect of 4000 IU/day for 48 weeks on the time to a severe asthma exacerbation or a viral induced exacerbation [39]. In this study, the initial mean blood levels were on the low side at 25 ng/ml: supplements caused a significant rise in blood levels.

Whether or not vitamin D supplements are beneficial in childhood remains controversial, and based on currently available studies, routine vitamin D supplementation is not recommended for children aged more than 2 years [40,41].

### Neuro-psychological disorders

The hypothesis that micronutrient deficiencies contribute to mood and neurological disorders is based on a rational physiological basis: iron and vitamin D have raised interest.

The autism spectrum disorder (ASD) is a neurodevelopmental disorder that affects several areas of mental development: uncertainty persists as to the efficacy of therapeutic diets [42]. Nevertheless, a retrospective survey of consumers who purchased Autism Nutrition Research Center products (ANRC-EP), a multi-micronutrient product for children or adults with ASD, enrolled 211 people including 85 children aged 5--15 years. The study showed significant benefits for a wide range of symptoms, and low adverse effects. Of note, no blood levels were available, and the supplements contained a combination of micronutrient delivering doses modestly above DRI [43].

A study including 430 children aged 7--10 years free of neurological disorders showed that trace element levels were linked to intellectual development and selective eating disorders: low blood levels were present in 20.3% for iron, 42.5% for zinc and 14% for copper levels, majority of children presenting more than one low levels [44^▪^].

A Thai RCT, including 52 participants with iron deficiency, tested ferrous iron supplements versus placebo for 12 weeks [45^▪▪^]: a significant improvement of ADHD symptoms was reported by the parents.

Attention-deficit/hyperactivity disorder (ADHD) is related to neurodevelopmental disorders potentially due to micronutrient deficits. The prevalence of ADHD has increased and despite pharmacological and psychological treatments, a substantial number of children with ADHD remain symptomatic [46]. A RCT including 74 children with ADHD tested a double-blind vitamin D intervention delivering (50 000 IU/week) plus magnesium (6 mg/kg/day) and showed a significant improvement of behavioural function and mental health. Of note, the children with mean age 9.1 years started with very low vitamin D levels (17 ng/ml), with a correction towards normal levels in the intervention group: the intervention and improved function was therefore associated with the correction of a deficit [46].

## FOOD FORTIFICATION AND SUPPLEMENTS

There is no controversy regarding the utility and efficacy of the food fortification programs to fight endemic deficiencies in low to middle-income countries [5,6], and milk fortification is a validated strategy in infants. But there is no consensus regarding the treatment of borderline status in high-income countries. Current data indicate that worldwide supplements are consumed by 24–38% of school-age children [47], confirming an analysis of the US National Health data base 2003–2006 [48], which showed that the prevalence of supplement use was 42% in 2–8 years. In this age group, there seemed to be limited inadequacies for other micronutrient intakes, while a higher prevalence of inadequate intakes of vitamins A, C and E was observed among 9 to 18-year-olds. In all age groups, supplement use increased the likelihood of intakes above the upper tolerable intake level for some micronutrients.

The study also showed the inadequacy of some supplements available on the market in different age groups [48]. Indeed, ‘more is not always better’, as Upper Limit (UL) may be exceeded, as stressed by the WHO, and may occur when different preventions programs overlap [49]. Commercial supplements are confronted the difficulty of different DRI/RDA over age: the products available for kids cover the needs in a highly variable way (Table 2). The German consumer organization gathered a large sample of products available over the counter or web [47], and analysed their content, which in some cases is problematic (too low or too high doses). Therefore, prescription of supplements should follow a careful dietary history, and address two very different situations: global malnutrition in which the intervention should be ‘nutrition’ with specific supplements oriented towards the most frequent deficiencies while integrating socioeconomic possibilities, that is, anaemia and iron deficiency, and the situation of picky eating habits of incomplete foods, or disease related deficiencies, where micronutrient supplements alone may be the best option. Choosing the optimal product will be based on their capacity to cover DRI without being excessive, and on the detection of a suboptimal level of intake of specific micronutrients.

**Table 2:** Examples of the compositions of three products available in Europe, chosen at random (no conflict of interest), and proposed for children 4 year and up (doses per unit) for the micronutrients listed in Table 1

Micronutrient	Age years	DRI	Supradyn junior 4+, Roche ^a^	Kids Multi Frank fruities ^b^	Orthomol junior C plus ^c^
Iodine (μg/day)	4–8 9–13 M+F 14–18 M+F	65 73 95	*0 zero*	50	50
Iron (mg/day)^d^	4–8 9–13 M+F 14–18 M 14–18 F	10 8 11 15	4,5	*0 zero*	6,0
Selenium (μg/day)	4–8 9–13 M+F 14–18 M+F	23 35 45	*0 zero*	*0 zero*	10
Zinc (mg/day)	4–8 9–13 M+F 14–18 M 14–18 F	4,0 7,0 8,8 7,3	4,8	*0 zero*	3,0
Vitamin A (μg/day, RE)	4–8 9–13 M 9–13 F 14–18 M 14–18 F	275 445 420 630 485	299	220	300
B9 folic acid (μg/day)	4–8 9–13 M+F 14–18 M+F	160 250 330	*0 zero*	*0 zero*	200
B12 Cobalamin (μg/day)	4–8 9–13 M+F 14–18 M+F	1,0 1,5 2,0	2,25	2.0	5,0
Vitamin C (mg/day)	4–8 9–13 M+F 14–18 M 14–18 F	22 39 63 56	75	*0 zero*	200
Vitamin D^d^ (μg/day) cholecalciferol	4–8 9–13 M+F 14–18 M+F	5 5 5	6	*0 zero*	10

A much more complete list is available from a German consumer organisation survey [47].

a Product includes B1, B2, niacin (B3), and recommends delivering 1–2 toffees/day 4–6 years and 3/day >6 years.

b Product also contains niacin, biotine (B7) and vitamin E.

c Product also contains vitamiB1, B2, B3, B5, B6, E and K and trace elements (Cu, Mn, Mo). Orthomol is a family business based in Langenfeld, North Rhine-Westphalia, Germany.

d RDA values for iron and cholecalciferol.

## CONCLUSION

The above review shows the importance of early diagnosis and detection of deficiency in children and adolescents, especially in families on vegetarian/strict vegan diets, or with socioeconomic difficulties. The detection of situations at risk, accompanied by objective determination of status appears very important. Studies in pregnant women and infants are not reported herein, but foetal and infant development are directly impacted by the mothers’ deficiencies.

The decision about the optimal strategy to apply requires considering the global context to the child, and the analysis of the available facilities surrounding the children. Systematic delivery of supplements includes a risk of excessive intakes. Multifaceted strategies (food fortification, education) to improve nutrition environment in different age groups is important in addressing the widespread problem of micronutrient deficiencies.

## Acknowledgements

*None.*

### Financial support and sponsorship

*None.*

### Conflicts of interest

*None.*

## REFERENCES AND RECOMMENDED READING

Papers of particular interest, published within the annual period of review, have been highlighted as:

▪ of special interest
▪▪ of outstanding interest

## References

[1] Requirements Institute of Medicine (IOM). Child and Adult Care Food Program: Aligning Dietary Guidance for All. Washington (DC): National Academies Press (US); 2011. 24983044

[2] EFSA. Overview on Dietary Reference Values for the EU population as derived by the EFSA Panel on Dietetic Products, Nutrition and Allergies (NDA). EFSA journal 2017. https://www.efsa.europa.eu/sites/default/files/assets/DRV_Summary_tables_jan_17.pdf.

[3] Taylor CM Emmett PM. Picky eating in children: causes and consequences. Proc Nutr Soc 2019; 78:161–169. 30392488 10.1017/S0029665118002586 PMC6398579

[4] Ara G Little DC Mamun AA. Factors affecting the micronutrient status of adolescent girls living in complex agro-aquatic ecological zones of Bangladesh. Sci Rep 2023; 13:6631. 37095307 10.1038/s41598-023-33636-8 PMC10126111

[5] World, Health, Organisation, WHO. Multiple micronutrient powders for point-of-use fortification of foods consumed by children 2–12 years of age. Geneva: WHO; Licence: CC BY-NC-SA 3.0 IGO; 2016.

[6] UNICEF, Section NaCD. Advancing large scale food fortification - UNICEF's vision and approach to persitent micronutrient deficiencies. https://unicef.org/media/110346/file/Advancing%20Large%20Scale%20Food%20Fortification.%20UNICEF's%20Vision%20and%20Approach.pdf. 2021. pp. 1-15.

[7] Cole CR. Preventing hidden hunger in children using micronutrient supplementation. J Pediatr 2012; 161:777–778. 22867986 10.1016/j.jpeds.2012.06.053

[8] Sichert-Hellert W Wenz G Kersting M. Vitamin intakes from supplements and fortified food in German children and adolescents: results from the DONALD study. J Nutr 2006; 136:1329–1333. 16614425 10.1093/jn/136.5.1329

[9] Domellof M Szitanyi P Simchowitz V. ESPGHAN/ESPEN/ESPR/CSPEN guidelines on pediatric parenteral nutrition: iron and trace minerals. Clin Nutr 2018; 37:2354–2359. 30078716 10.1016/j.clnu.2018.06.949

[10] Hardy G Wong T Morrissey H. Parenteral provision of micronutrients to pediatric patients: an international Expert Consensus Paper. JPEN J Parenter Enteral Nutr 2020; 44: (Suppl 2): S5–S23. 10.1002/jpen.1990 32767589

[11] Berger MM Shenkin A Schweinlin A. ESPEN micronutrient guideline. Clin Nutr 2022; 41:1357–1424. 35365361 10.1016/j.clnu.2022.02.015

[12] UNICEF-Uganda. Key practice: Micronutrients for children, adolescents and women - why they are important for your family. https://www.unicef.org/uganda/key-practice-micronutrients-children-adolescents-and-women. 2024.

[13] Suthutvoravut U Abiodun PO Chomtho S. Composition of follow-up formula for young children aged 12-36 months: recommendations of an International Expert Group Coordinated by the Nutrition Association of Thailand and the Early Nutrition Academy. Ann Nutr Metab 2015; 67:119–132. 26360877 10.1159/000438495

[14] Tam E Keats EC Rind F. Micronutrient supplementation and fortification interventions on health and development outcomes among children under-five in low- and middle-income countries: a systematic review and meta-analysis. Nutrients 2020; 12:289. 31973225 10.3390/nu12020289 PMC7071447

[15] Allen LH. Zinc and micronutrient supplements for children. Am J Clin Nutr 1998; 68:495S–498S. 9701167 10.1093/ajcn/68.2.495S

[16] Doom JR Richards B Caballero G. Infant iron deficiency and iron supplementation predict adolescent internalizing, externalizing, and social problems. J Pediatr 2018; 195:199–205. 29395182 10.1016/j.jpeds.2017.12.008 PMC5869133

[17▪] Trapani S Rubino C Indolfi G Lionetti P. A narrative review on pediatric scurvy: the last twenty years. Nutrients 2022; 14:684. 35277043 10.3390/nu14030684 PMC8840722

[18▪] Hargreaves D Mates E Menon P. Strategies and interventions for healthy adolescent growth, nutrition, and development. Lancet 2022; 399:198–210. 34856192 10.1016/S0140-6736(21)01593-2

[19▪▪] Chao HC Lu JJ Yang CY. Serum trace element levels and their correlation with picky eating behavior, development, and physical activity in early childhood. Nutrients 2021; 13:2295. 34371805 10.3390/nu13072295 PMC8308333

[20] Kuriyan R Thankachan P Selvam S. The effects of regular consumption of a multiple micronutrient fortified milk beverage on the micronutrient status of school children and on their mental and physical performance. Clin Nutr 2016; 35:190–198. 25746819 10.1016/j.clnu.2015.02.001

[21] Nawab F Fatima S Nazli R. Micronutrient status and energy intake in moderate acute malnourished children after intake of high energy nutritional supplements for four weeks: a randomized controlled study. J Ayub Med Coll Abbottabad 2022; 34:239–246. 35576279 10.55519/JAMC-02-9106

[22▪] Ning Y Hu M Chen S. Investigation of selenium nutritional status and dietary pattern among children in Kashin-Beck disease endemic areas in Shaanxi Province, China using duplicate portion sampling method. Environ Int 2022; 164:107255. 35561595 10.1016/j.envint.2022.107255

[23▪] Chouraqui JP. Risk assessment of micronutrients deficiency in vegetarian or vegan children: not so obvious. Nutrients 2023; 15:2129. 37432244 10.3390/nu15092129 PMC10180846

[24▪] Svetnicka M Henikova M Selinger E. Prevalence of iodine deficiency among vegan compared to vegetarian and omnivore children in the Czech Republic: cross-sectional study. Eur J Clin Nutr 2023; 77:1061–1070. 37488261 10.1038/s41430-023-01312-9 PMC10630131

[25] Svetnicka M Sigal A Selinger E. Cross-sectional study of the prevalence of cobalamin deficiency and Vitamin B12 supplementation habits among vegetarian and vegan children in the Czech Republic. Nutrients 2022; 14:535. 35276893 10.3390/nu14030535 PMC8838497

[26] Lemale J Mas E Jung C. Vegan diet in children and adolescents. Recommendations from the French-speaking Pediatric Hepatology, Gastroenterology and Nutrition Group (GFHGNP). Arch Pediatr 2019; 26:442–450. 31615715 10.1016/j.arcped.2019.09.001

[27] Stevens GA Finucane MM De-Regil LM. Global, regional, and national trends in haemoglobin concentration and prevalence of total and severe anaemia in children and pregnant and nonpregnant women for 1995-2011: a systematic analysis of population-representative data. Lancet Glob Health 2013; 1:e16–e25. 25103581 10.1016/S2214-109X(13)70001-9 PMC4547326

[28▪] Yusufu I Cliffer IR Yussuf MH. Factors associated with anemia among school-going adolescents aged 10-17 years in Zanzibar, Tanzania: a cross sectional study. BMC Public Health 2023; 23:1814. 37723498 10.1186/s12889-023-16611-w PMC10508009

[29] Handiso YH Belachew T Abuye C. A community-based randomized controlled trial providing weekly iron-folic acid supplementation increased serum- ferritin, -folate and hemoglobin concentration of adolescent girls in southern Ethiopia. Sci Rep 2021; 11:9646. 33958657 10.1038/s41598-021-89115-5 PMC8102612

[30▪] Gupta A Kant S Ramakrishnan L. Impact of daily-supervised administration of a package of iron and folic acid and vitamin B(12) on hemoglobin levels among adolescent girls (12-19 years): a cluster randomized control trial. Eur J Clin Nutr 2021; 75:1588–1597. 33828241 10.1038/s41430-021-00878-6

[31▪] Hoang NTD Orellana L Gibson RS. Multiple micronutrient supplementation improves micronutrient status in primary school children in Hai Phong City, Vietnam: a randomised controlled trial. Sci Rep 2021; 11:3728. 33580103 10.1038/s41598-021-83129-9 PMC7881239

[32] Tandon R Thacker J Pandya U. Parenteral vs oral Vitamin B12 in children with nutritional macrocytic anemia: a randomized controlled trial. Indian Pediatr 2022; 59:683–687. 35642923

[33▪▪] Black MM Fernandez-Rao S Nair KM. A randomized multiple micronutrient powder point-of-use fortification trial implemented in Indian preschools increases expressive language and reduces anemia and iron deficiency. J Nutr 2021; 151:2029–2042. 33880548 10.1093/jn/nxab066 PMC8245888

[34▪▪] Bassouni R Soliman M Hussein LA. Development and evaluating the biopotency of ready to eat liver meat balls in fighting anaemia and vitamin A deficiency, improving selected nutritional biochemical indicators and promoting the cognitive function among mildly anaemic Egyptian children aged 3-9 years. Public Health Nutr 2022; 25:3182–3194. 35451359 10.1017/S1368980022000970 PMC9991726

[35] Berglund SK Domellof M. Iron deficiency in infancy: current insights. Curr Opin Clin Nutr Metab Care 2021; 24:240–245. 33656466 10.1097/MCO.0000000000000749

[36] Aslan N. A case of iron intoxication treated by plasmapheresis. Indian J Pediatr 2022; 89:315. 34826057 10.1007/s12098-021-04025-8

[37▪] Ganmaa D Bromage S Khudyakov P. Influence of Vitamin D supplementation on growth, body composition, and pubertal development among school-aged children in an area with a high prevalence of Vitamin D deficiency: a randomized clinical trial. JAMA Pediatr 2023; 177:32–41. 36441522 10.1001/jamapediatrics.2022.4581 PMC9706398

[38▪] Tamara L Kartasasmita CB Alam A Gurnida DA. Effects of Vitamin D supplementation on resolution of fever and cough in children with pulmonary tuberculosis: a randomized double-blind controlled trial in Indonesia. J Glob Health 2022; 12:04015. 35198149 10.7189/jogh.12.04015 PMC8855907

[39] Forno E Bacharier LB Phipatanakul W. Effect of Vitamin D3 supplementation on severe asthma exacerbations in children with asthma and low Vitamin D levels: the VDKA randomized clinical trial. JAMA 2020; 324:752–760. 32840597 10.1001/jama.2020.12384 PMC7448830

[40] Reinehr T Schnabel D Wabitsch M. Vitamin D supplementation after the second year of life: joint position of the Committee on Nutrition, German Society for Pediatric and Adolescent Medicine (DGKJ e.V.), and the German Society for Pediatric Endocrinology and Diabetology (DGKED e.V.). Mol Cell Pediatr 2019; 6:3. 31062205 10.1186/s40348-019-0090-0 PMC6502918

[41] Braegger C Campoy C Colomb V. Vitamin D in the healthy European paediatric population. J Pediatr Gastroenterol Nutr 2013; 56:692–701. 23708639 10.1097/MPG.0b013e31828f3c05

[42] Onal S Sachadyn-Krol M Kostecka M. A review of the nutritional approach and the role of dietary components in children with autism spectrum disorders in light of the latest scientific research. Nutrients 2023; 15:4852. 38068711 10.3390/nu15234852 PMC10708497

[43] Adams JB Kirby J Audhya T. Vitamin/mineral/micronutrient supplement for autism spectrum disorders: a research survey. BMC Pediatr 2022; 22:590. 36229781 10.1186/s12887-022-03628-0 PMC9558401

[44▪] Saati AA Adly HM. Assessing the correlation between blood trace element concentrations, picky eating habits, and intelligence quotient in school-aged children. Children (Basel) 2023; 10:1249. 37508746 10.3390/children10071249 PMC10378148

[45▪▪] Pongpitakdamrong A Chirdkiatgumchai V Ruangdaraganon N. Effect of iron supplementation in children with attention-deficit/hyperactivity disorder and iron deficiency: a randomized controlled trial. J Dev Behav Pediatr 2022; 43:80–86. 34313619 10.1097/DBP.0000000000000993

[46] Hemamy M Pahlavani N Amanollahi A. The effect of vitamin D and magnesium supplementation on the mental health status of attention-deficit hyperactive children: a randomized controlled trial. BMC Pediatr 2021; 21:178. 33865361 10.1186/s12887-021-02631-1 PMC8052751

[47] German Consumer Organisation. Food supplements for children - market check of the consumer centres. https://www.verbraucherzentrale.de. 2019. pp. https://www.klartext-nahrungsergaenzung.de/sites/default/files/2019-08/MarktcheckNEM-fuer-Kinder-BMEL_Englisch-2019.pdf.

[48] Bailey RL Fulgoni VL 3rd Keast DR. Do dietary supplements improve micronutrient sufficiency in children and adolescents? J Pediatr 2012; 161:837–842. 22717218 10.1016/j.jpeds.2012.05.009 PMC3477257

[49] Pike V Zlotkin S. Excess micronutrient intake: defining toxic effects and upper limits in vulnerable populations. Ann N Y Acad Sci 2019; 1446:21–43. 30569544 10.1111/nyas.13993

